# *MxCuBE*: a synchrotron beamline control environment customized for macromolecular crystallography experiments

**DOI:** 10.1107/S0909049510020005

**Published:** 2010-07-13

**Authors:** José Gabadinho, Antonia Beteva, Matias Guijarro, Vicente Rey-Bakaikoa, Darren Spruce, Matthew W. Bowler, Sandor Brockhauser, David Flot, Elspeth J. Gordon, David R. Hall, Bernard Lavault, Andrew A. McCarthy, Joanne McCarthy, Edward Mitchell, Stéphanie Monaco, Christoph Mueller-Dieckmann, Didier Nurizzo, Raimond B. G. Ravelli, Xavier Thibault, Martin A. Walsh, Gordon A. Leonard, Sean M. McSweeney

**Affiliations:** a European Synchrotron Radiation Facility, 6 rue Jules Horowitz, BP 220, 38043 Grenoble, France; b European Molecular Biology Laboratory, 6 rue Jules Horowitz, BP 181, 38042 Grenoble, France; c MRC-France (BM14), c/o ESRF, BP 220, 38043 Grenoble, France

**Keywords:** automation, macromolecular crystallography, synchrotron beamline control, graphical user interface

## Abstract

The design and features of a beamline control software system for macromolecular crystallography (MX) experiments developed at the European Synchrotron Radiation Facility (ESRF) are described. This system, *MxCuBE*, allows users to easily and simply interact with beamline hardware components and provides automated routines for common tasks in the operation of a synchrotron beamline dedicated to experiments in MX. Additional functionality is provided through intuitive interfaces that enable the assessment of the diffraction characteristics of samples, experiment planning, automatic data collection and the on-line collection and analysis of X-ray emission spectra. The software can be run in a tandem client-server mode that allows for remote control and relevant experimental parameters and results are automatically logged in a relational database, ISPyB. *MxCuBE* is modular, flexible and extensible and is currently deployed on eight macromolecular crystallography beamlines at the ESRF. Additionally, the software is installed at MAX-lab beamline I911-3 and at BESSY beamline BL14.1.

## Abstract 2

*MxCuBE* is a beamline control environment optimized for the needs of macromolecular crystallography. This paper describes the design of the software and the features that *MxCuBE* currently provides.

## 1. Introduction

The European Synchrotron Radiation Facility (ESRF) Structural Biology Group runs six beamlines for macromolecular crystallography (MX). These form a comprehensive suite of resources that cater to the various requirements that MX samples demand. Some of the facilities have been operational for more than a decade (Wakatsuki *et al.*, 1998 ▶; McCarthy *et al.*, 2009 ▶), while the newest members have been in service for only a few years (Nurizzo *et al.*, 2006 ▶; Flot *et al.*, 2010 ▶). Since the first beamline in this suite was commissioned, the number of macromolecular crystal structures elucidated per year using diffraction data collected at the ESRF has increased more than 20-fold. This increase in productivity has arisen owing to a number of initiatives which have augmented both the rate at which crystals have become available and the rate at which diffraction data can be collected, analysed and phased.

Since the construction of the first synchrotron beamlines dedicated to MX, the pressure for productive use of beam time has been high. In order to allow experimenters unfamiliar with beamline control to easily use such resources, graphical user interfaces (GUIs) were developed to simplify the use of these facilities (Kinder *et al.*, 1996 ▶; Skinner & Sweet, 1998 ▶). The evolution of these programs has led to an increasing degree of sophistication (McPhillips *et al.*, 2002 ▶; Skinner *et al.*, 2006 ▶) and the software now used in GUI construction allows the exploitation of control services distributed over many computers and the linking of experimental parameters to information storage systems.

At the ESRF the beamline control GUI *MxCuBE* is the successor to the *ProDC* interface (Arzt *et al.*, 2005 ▶) and builds on work started during the construction of ID23-1 (Nurizzo *et al.*, 2006 ▶) to provide a consistent user environment that enables the routine use of automated beamlines coupled with robotic sample changers. *MxCuBE* presents, as far as is practicable, the same options, in the same way on different beamlines. Variations in X-ray beam characteristics, optical layout and experimental hutch hardware are dealt with by modifying the configuration of *MxCuBE* or the low-level software called by a particular *MxCuBE* functionality. *MxCuBE* also provides access to ancillary tools including the assessment of the diffraction characteristics of samples, experiment planning and automatic data collection, and the on-line analysis of X-ray emission spectra that add value to MX experiments.

In this paper we describe the design of the software, which ensures that the *MxCuBE* environment will be adaptable to emerging needs, and the features which *MxCuBE* currently provides.

## 2. Design of the software

In general, software for beamline control follows a common layout: the GUI functionality is used both to hide underlying beamline specific interactions and to offer parameters and methods for data collection. The approach taken in *MxCuBE* follows this general paradigm with low-level motor control hidden from the experimenter and complex analysis protocols provided *via* functions activated from the GUI.

**Figure 1:**
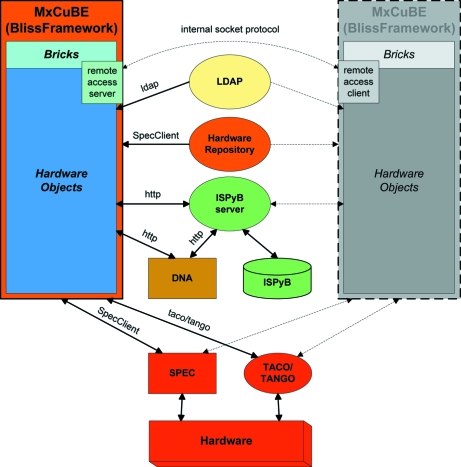
Beamline control within the BlissFramework architecture. The *MxCuBE* application (orange) is composed of bricks, hardware objects and the Hardware Repository (also part of the Framework). Also shown are the LDAP server, the ISPyB server and database and the DNA Expert System. Hardware and its control systems are shown in red. For remote access further instances of *MxCuBE* are created (here a single further instance is shown in grey) and connected to the server instance (orange) that is displayed and started on the beamline control workstation at the ESRF.

The application (Fig. 1 ▶) is built using the BlissFramework (Guijarro *et al.*, 2004 ▶), an internal software development platform created at the ESRF. The main goal of the BlissFramework is to ease the creation of graphical applications for beamline instrumentation control by providing services to allow the simplified integration and configuration of new graphical components. With a fast development cycle in mind, such applications are created by combining several self-contained graphical widgets (bricks, §2.2), each representing a particular view of, or mode of interaction with, a hardware object that controls a physical instrument.

*MxCuBE* was the first application to take advantage of this platform. It has been developed in Python (http://www.python.org/) with the Qt library (Trolltech; http://trolltech.com/products/; Riverbank Computing; http://www.riverbankcomputing.co.uk/software/pyqt/) as its graphics toolkit. Taking a modular approach, *MxCuBE* separates control (hardware objects, §2.1) from graphical representation (bricks, §2.2). Low-level control is achieved *via* connection to TACO or TANGO servers (http://www.tango-controls.org/) and to the macro language SPEC (Certified Scientific Software; http://www.certif.com/).

### 2.1. Hardware objects

Hardware objects are self-contained pieces of software code which link graphical bricks (§2.2) to the underlying instrumentation control software. Their purpose is to provide a way for an application’s graphical widgets to interact with an instrument (*e.g.* sample changer) or motor (*e.g.* monochromator rotation axis). It is common for one hardware object to interact with more than one piece of underlying control software in order to implement a workflow which uses multiple hardware components or procedures. The BlissFramework provides the methods for creating hardware objects which are configured using eXtensible Markup Language (XML) files loaded through a server. Only one instance of a hardware object runs for a given piece of instrumentation and this instance is available to handle requests made by other hardware objects or bricks. A hardware object provides a Python application programming interface (API) and handles asynchronous communication between other hardware objects or bricks by emitting signals.

### 2.2. Bricks

Bricks are the individual graphical objects used to make up the GUI seen by a user. They are normally connected to hardware objects in order to allow the display of information and motor control. There are no restricted relationships: one hardware object can have multiple bricks as graphical views (each brick with a different purpose) or a single brick can interact with several hardware objects. Bricks are made configurable by defining properties which are set using the application builder (§2.3). The connection of a brick to a hardware object is configured *via* a series of properties. This mechanism makes it easy to change the application’s configuration after deployment. Bricks also communicate with each other using mechanisms defined in the brick’s code file and set with the application builder. This approach was chosen to encourage the creation of small function-specific bricks rather than large multi-functional monolithic bricks that are much harder to maintain and develop. The developer then creates a data flow by connecting these smaller bricks together.

### 2.3. Application builder

The application builder is a graphical editor developed to create a beamline control GUI and can be launched in ‘run’ or ‘design’ modes. In the former, the application is executed as defined in its configuration file. If launched in design mode, extra windows are displayed that allow the GUI to be modified: bricks can be added or removed, their connections modified and some characteristics of the appearance changed. Linking the run and design modes in this fashion allows for the rapid development and testing of new functionality and GUI layouts.

### 2.4. Hardware repository server

Underlying all the components of the BlissFramework is the Hardware Repository Server which contains all the configuration information necessary for a hardware object to operate a physical device. By creating a shared configuration pool, multiple BlissFramework applications, for example an instance of *MxCuBE* running in the experiment control cabin and a stripped-down version running in the experimental hutch, can interact with identical hardware objects as both access the same hardware repository. Communication with a hardware repository server is *via* sockets over which a filename argument (corresponding to the XML configuration of a hardware object) is sent and the contents of that file returned.

## 3. The *MxCuBE* GUI

**Figure 2:**
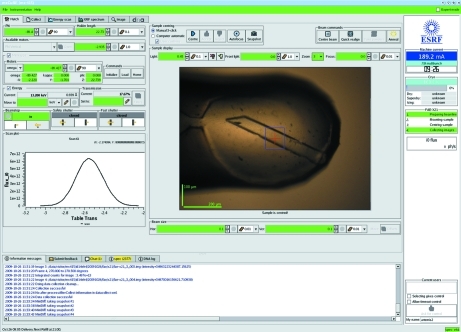
The Hutch tab of the *MxCuBE* GUI. Expert mode is accessed by clicking the check box in the top right of the GUI. The plot window shows a scan of beam intensity *versus* horizontal translation of the experiment table carried out as part of the ‘quick realign’ procedure. The image-viewing brick shows a sample after centring in the X-ray beam (blue square) using the 3-click option.

To simplify beamline control, bricks are clustered into a series of tabs designed to allow the execution of various different tasks associated with a MX experiment. However, some functionality and information display is available in all tabs. A bar at the top of the GUI, colour-coded according to user status (orange: not logged in; yellow: logged in but not in control; green: logged in and in control), gives access to an ‘instrumentation’ pull-down menu and to the password-protected ‘Expert’ mode of the GUI. The former allows user-defined positioning (*i.e.* in or out) of various hardware. The latter gives access to specialized motors (*i.e.* for slit offset, goniometer and beamstop translation axes) or functionality (*i.e.* detector distance and beam centre calibration routines) and is normally accessed only by beamline staff. All tabs of *MxCuBE* also display the status of the storage ring [current (mA) and lifetime (hours)], the cryo-stream [temperature (K) and liquid nitrogen level], the current status of the beamline and the brick allowing remote control of a beamline. Across the bottom of the *MxCuBE* window a series of tabs provide: the display of information messages and beamline events (shown in Fig. 2 ▶); a simple e-mail feedback form; a chat/instant messaging server used principally during remote-access experiments (§3.5); a means of directly interacting with the SPEC middle control layer; and the log files from DNA [automateD collectioN of datA, an expert system that allows for the screening of sample diffraction quality, the calculation of data collection strategies and the automatic collection of diffraction data (§3.4; Leslie *et al.*, 2002 ▶)].

*MxCuBE* displays a video feed of the sample position *via* the Camera brick in the ‘Hutch tab’ (§3.1). This is fed with images by the Camera Hardware Object which sits on top of a Tango-based middle-ware device server for the on-axis viewer CCD cameras (Prosilica GC655C) installed on the ESRF MX beamlines. Images (659 × 493 pixels; pixel size 9 µm × 9 µm) from the camera are submitted to the camera brick in Bayer format, interpolated RGB and decompressed using the Qub library (ESRF in-house development) and displayed at a rate of 15–25 frames per second depending on how the CCD camera is linked to the control computer. The Tango device server is coupled with an associated Calculation Server that allows image analysis, including the determination of the ‘centre of mass’ that is used in the ‘Centre beam’ option provided in the Hutch tab (§3.1) of *MxCuBE*. There are two regular clients for the camera device server: the *MxCuBE* instance running in the control cabin and a stripped-down version [comprising only the Hutch tab (§3.1) and sample changer brick (§3.2)] running in the experimental hutch. During remote-access experiments (§3.5) this rises to three clients. Monitoring of the device server operation has shown no significant decrease in performance when serving up to at least four clients with current hardware.

Before any experimental session can start, the experimenter is required to log in using an experiment identifier and password contained in the ESRF LDAP (Lightweight Directory Access Protocol) server. The requirement to log in ensures appropriate use of the beamline and establishes, *via* the ISPyB database (Beteva *et al.*, 2006 ▶), a link between experiment identifier and sample safety information. The act of logging in also activates the electronic data collection notebook services provided within ISPyB. Before any given experimental session, users have the option of uploading to ISPyB information concerning the individual crystals to be studied in the session. Essential information for each crystal comprises the sample name, its protein acronym and the sample changer basket in which it is contained. Additional information includes space group and unit-cell parameters, required resolution, sample barcode and position in the basket. The latter two pieces of information are mutually exclusive. If such information is supplied in ISPyB, a list of samples to be studied in the current experimental session is provided in *MxCuBE*. This list appears in a ‘sample area’ in both the Hutch (§3.3) and ‘DNA’ (§3.4) tabs of the GUI. Highlighting a particular sample causes *MxCuBE* to set default values for directory and file names for data collection based on a convention using the sample’s name and acronym. Automatic increments in ‘run number’ serve to avoid overwriting images with the same filename prefix.

### 3.1. The Hutch tab

The Hutch tab (Fig. 2 ▶) provides access to the functionality necessary for preparing an experiment. The motors that are most commonly used in establishing the experimental configuration have dedicated bricks, with their status being indicated through the use of a colour-coded scheme: green for normal, yellow for moving, red for alarm, and grey for disabled.

A number of higher-level beamline control operations are also provided by bricks in the Hutch tab. ‘Quick realign’ triggers the alignment of optics and/or experimental hutch components and ensures that the X-ray beam delivered to the sample is optimized for intensity at the current working energy. The particular sequence of events instigated is specific to the beamline where the instance of *MxCuBE* is running. When required, and once a scintillator crystal has been placed at the sample position, ‘Centre beam’ uses a peak-finding algorithm built into the video server to locate the centre of the X-ray beam and move the experimental table and slits such that sample position and beam intersect.

To view and align samples the Hutch tab displays a video feed showing the sample position. Two different methods of sample alignment are available: 3-click and automatic. In the 3-click option the sample is rotated in ϕ by 3 × 90°, the crystal centre identified *via* a mouse click after each 90° rotation and the motor movements required to place the sample in the X-ray beam calculated and implemented. Fully automatic crystal centring is achieved using image-processing algorithms in C3D (Lavault *et al.*, 2006 ▶). After both 3-click and auto-centring, a manual approval or rejection of the result is available. Upon acceptance, four snapshot images are taken, each separated by 90° in ϕ. These images are transferred to an archive and made accessible *via* the ISPyB database (Beteva *et al.*, 2006 ▶).

Other general features available *via* Hutch tab bricks include: changing the size and intensity of the X-ray beam incident on the sample (the ‘slit’ and ‘transmission’ bricks), automatic annealing of the sample currently mounted (Giraud *et al.*, 2009 ▶), opening and closing of beamline shutters (for beam visualization on scintillator crystals) and in/out positioning of the beamstop (fine alignment of the beamstop is available in ‘Expert’ mode). The motor positions of the add-on mini-kappa goniometer head (McCarthy *et al.*, 2009 ▶) are also monitored and controlled using the Hutch tab. For diffractometers (*i.e.* MD2; Cusack *et al.*, 1998 ▶) where the size of the X-ray beam incident on the sample can be modified using apertures, these can also be inserted and removed *via* a brick in this tab.

### 3.2. The Collect tab

**Figure 3:**
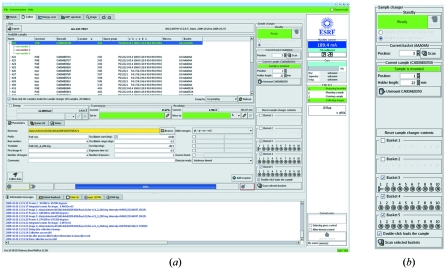
(*a*) The Collect tab of the *MxCuBE* GUI. The sample area in the top portion of the GUI shows information contained in the ISPyB LIMS (sample name, protein acronym, barcode, space group, unit-cell dimensions *etc.*) for the samples contained in the SC3 sample changer. (*b*) The sample-changer brick embedded in the Collect tab. The two-dimensional barcodes of the SPINE standard pins contained in the SC3 are read by clicking ‘scan selected baskets’. The sample currently mounted is flagged in both the sample changer brick and in the sample area (shown in bold green). In this figure MAD/SAD data collection (see text) is not activated.

The Collect tab (Fig. 3*a* ▶) is perhaps the most important aspect of the *MxCuBE* GUI as both sample selection and data collection are performed from within this interface. Here, simple features ease beamline use. On tunable beamlines the operating energy can be specified either in energy (keV) or wavelength (Å) according to the experimenter’s preference. The diffraction geometry can be defined by either sample-to-detector distance, by a desired diffraction resolution (calculated using the minimum dimension of the detector) or automatically generated from the diffraction properties of the sample (DNA tab, §3.4).

Other data collection parameters (ϕ_start_, Δϕ, exposure time, number of images) can be entered into the tab manually or by running DNA (§3.4). Before a data collection can start, these values are validated by a SPEC macro to ensure consistency with instrument capabilities. In the case of invalid collection parameters a dialogue box explaining the conflict, and preventing data collection, appears. If the collection parameters are valid, a confirmation dialogue box pops up, previewing the filenames of the oscillation images to be collected and highlighting the names of files that already exist.

For MAD or SAD experiments, X-ray energies for the collection of peak (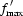), inflection point (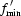) and remote data sets are received from the ‘Energy Scan’ tab (see §3.3) after an absorption-edge scan is carried out. The number, energy and order of datasets to be collected are then confirmed in the Collect tab. Datasets are collected in the order defined and diffraction images are stored in sub-directories named, by default, according to the corresponding dataset energy. These sub-directory names are also user-configurable. A further option allowing inverse-beam data collection is available; this is done by checking the appropriate box and specifying the angular range of the wedges to be collected at ϕ and ϕ + 180°.

An important feature is the concept of queued data collection. Here, instead of initiating data collection directly, the parameters are stored for future use. This allows both for the implementation of multi-sweep data collection strategies and for complete datasets to be built up from multiple partial datasets collected at different positions within a crystal. For the latter option, various positions in the crystal are defined using the 3-click centring option (§3.1). After each centring operation, data collection parameters are entered into the queue. On launching the data collection, each partial data set is collected, in its turn, from the position in the crystal previously defined and stored by *MxCuBE*.

The option of ‘helical’ data collection (Flot *et al.*, 2010 ▶), particularly powerful when crystals are much larger than the incident X-ray beam size, is activated and controlled *via* a series of SPEC commands. Start and end positions are chosen by clicking on points on the crystal in the viewing brick in the Hutch tab and data collection is then initiated as normal. During a helical data collection the crystal is automatically translated between the two pre-defined points so as to gradually move a fresh part of the crystal into the X-ray beam. The time that each point spends in the X-ray beam is calculated automatically as a function of the distance between the two user-defined positions and the total rotation angle required during the data collection. The amount of data collected from each point is therefore constant. With helical data collection the X-ray dose received by the crystal is evenly distributed over the whole of the crystal segment chosen, rather than focused on one particular position; therefore longer exposure times per image are possible before radiation damage occurs. When helical data collection is activated, an information/warning message is displayed to users before data collection starts.

A basic ‘Image’ tab in *MxCuBE* (not described here) allows the display and inspection of individual diffraction images. However, this functionality is currently almost invariably carried out by launching the much more feature-rich *ADXV* image-viewing software (Area Detector Systems Corporation; Szebenyi *et al.*, 1997 ▶; http://www.scripps.edu/~arvai/adxv.html) on the beamline control computer. Such is the speed of data collection on modern synchrotron-based MX resources that, even using *ADXV*, detailed inspection of diffraction images is performed, in the majority of cases, only pre- and post-data-collection. This being the case, emphasis in *MxCuBE* has turned to the provision of tools (see §3.4, the DNA tab) that allow the rapid determination of crystal diffraction characteristics, the calculation and execution of data collection strategies and the on-line integration and analysis of the resulting intensity data, the latter producing a better idea of diffraction data quality than any visual inspection of diffraction images, no matter what software is used.

The Collect tab is also used to control the SC3 sample changers (Cipriani *et al.*, 2006 ▶) installed on the ESRF’s MX beamlines (Fig. 3*b* ▶). In addition to loading/unloading operations, features are incorporated for the scanning and display of the barcodes of samples currently in the sample changer. These barcodes form an integral part of the SPINE standard (Cipriani *et al.*, 2006 ▶) to which sample holders must conform if the SC3 is being used. Barcodes are displayed, along with sample information and sample changer position, in the sample area of both the Collect and DNA tabs. Mounting/unmounting of samples can be initiated either by clicking on a particular sample in the sample list, choosing sample changer position in the boxes provided or by clicking on a sample changer position in the matrix that is also provided in the tab. Icons in the matrix display the status of all sample changer positions, which are: sample present and barcode read or supplied *via* ISPyB (barcode icon); sample present but barcode not read or not supplied (filled white circle); no sample present (empty circle); unknown (question mark). When a sample is loaded a pin icon appears at the relevant position in the matrix and the relevant sample is highlighted in the sample list.

### 3.3. Energy scans and X-ray fluorescence spectra

**Figure 4:**
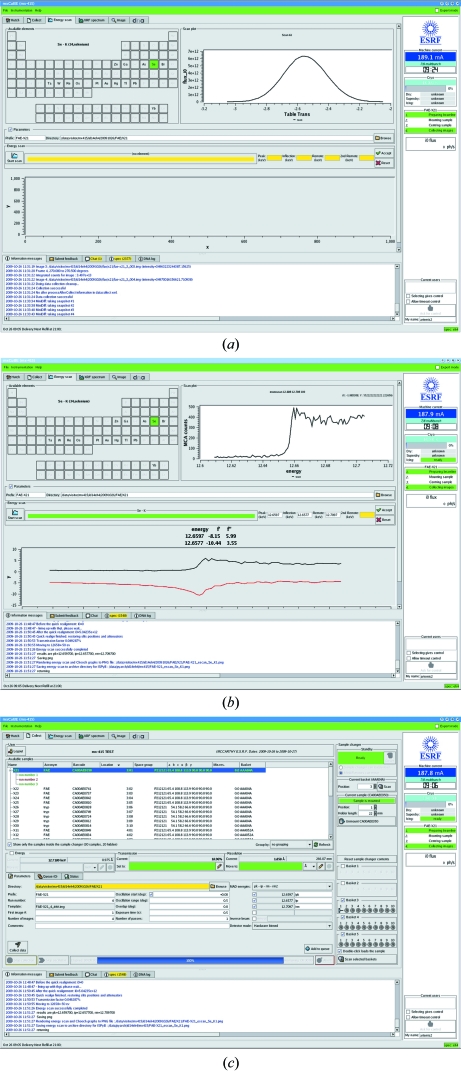
Absorption-edge scans in *MxCuBE*. (*a*) A scan is launched by clicking on an element in the periodic table in the ‘Energy scans’ tab. (*b*) Following a scan, the spectrum is analysed using *CHOOCH* (Evans & Pettifer, 2001 ▶), plots of *f*′ and *f*′′ against photon energy displayed and energies for the collection of peak, inflection point and remote wavelengths suggested. (*c*) Accepting the suggestions for the energies at which data are to be collected activates MAD/SAD data collection in the Collect tab. Here, the number and order of collection of datasets can be defined.

Absorption-edge or XANES (X-ray absorption near-edge structure) scans are performed in the Energy Scan tab (Fig. 4 ▶); the user can choose which edge to scan (for *L* edges the *L*_III_ edge only is available) by selecting the desired element from the periodic table displayed (only elements for which an absorption edge is accessible are highlighted). All raw data, plot and results files produced are stored under the chosen directory and with a common filename prefix, which is also entered in this tab.

Scans are performed according to principles established empirically (Arzt *et al.*, 2005 ▶). The resulting spectrum is analysed using *CHOOCH* (Evans & Pettifer, 2001 ▶) and plots of *f*′ and *f*′′ against energy are displayed. Energies for the collection of inflection point, peak and remote data sets are also presented (the latter is by default taken as 50 eV above the energy of the peak of the absorption edge). Accepting one or more of these energies activates the multi-wavelength data collection mode in the Collect tab (§3.2). At the end of the XANES scan procedure, the raw data file and the *CHOOCH*-transformed spectrum are archived and referenced in the ISPyB database.

**Figure 5:**
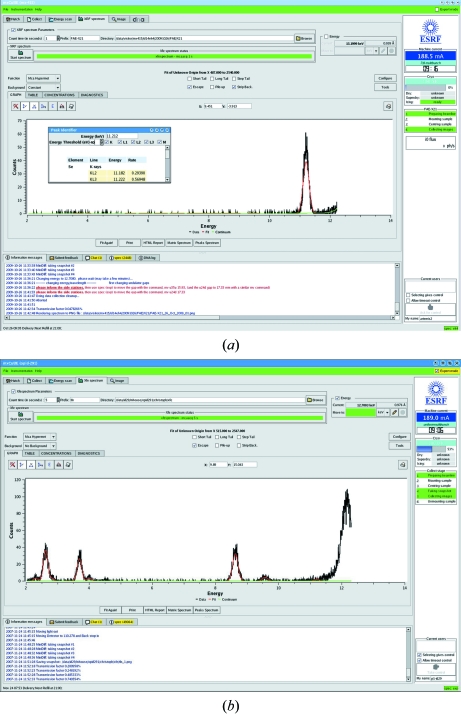
Analysis of X-ray-induced fluorescence (XRF) spectra in *MxCuBE*. (*a*) The spectrum from a crystal (see Fig. 2 ▶) of a selenomethionyl derivative of the feruloyl esterase module of xylanase 10B from *Clostridium thermocellum* (Tarbouriech *et al.*, 2005 ▶). The displayed spectrum is truncated so that peaks owing to Compton and Rayleigh scattering by the sample of the incident X-ray beam are not shown. Clicking on a peak in the spectrum allows identification of elements *via* a pop-up window. (*b*) The XRF spectrum from a crystal of thermolysin from *Bacillus thermoproteolyticus* (Mueller-Dieckmann *et al.*, 2007 ▶) showing the presence of chloride (*K*α emission line, ∼2.6 keV), potassium (*K*α, *K*β emission lines, ∼3.7 keV and ∼4.0 keV, respectively) and zinc (*K*α, *K*β emission lines, ∼8.6 keV and ∼9.6 keV, respectively). The peak at ∼12.2 keV is due to Compton scattering by the sample of the incident X-ray beam. The *K*α emission line of sulfur (∼2.3 keV) is also visible in this spectrum.

The collection and analysis of X-ray fluorescence (XRF) spectra is available *via* the ‘XRF Spectrum’ tab (Leonard *et al.*, 2009 ▶; Fig. 5 ▶). To perform the analysis a directory, filename prefix and measurement time are selected before clicking on the ‘Start spectrum’ button. The resulting fluorescence spectrum is displayed along with an analysis of the elements contributing to the spectrum. At the end of the procedure, both the XRF spectrum and an html report (summarizing the elements for which emission lines are identified) are archived and made available in ISPyB.

### 3.4. The DNA tab

The DNA system for automated sample evaluation and data collection (Leslie *et al.*, 2002 ▶) is a key part of the options offered to users of the ESRF MX beamlines. As DNA has its own GUI we have implemented two methods of interaction with *MxCuBE*. The first of these is *via* the DNA GUI (Leslie *et al.*, 2002 ▶). In the second, the DNA server is controlled directly from *MxCuBE*.

When using the DNA GUI the determination of sample changer contents (*i.e.* barcode scanning) and the subsequent extraction of sample information from ISPyB can be initiated from either the DNA GUI itself or from *MxCuBE* (§3.2). This information is then displayed in both the *MxCuBE* and the DNA GUIs. Two modes of operation are then possible. In a so-called ‘single shot’ mode individual samples are loaded and centred using *MxCuBE* (see above). Sample evaluation, including calculation of a data collection strategy, is then performed as described previously (Leslie *et al.*, 2002 ▶) and, if desired, data automatically collected. The on-line integration of diffraction images and analysis of the resulting intensity data, using *MOSFLM* (Leslie, 1992 ▶) and *SCALA* (Evans, 2006 ▶), respectively, is also available. In this mode *MxCuBE* acts as a slave to DNA. Data collection is controlled from the DNA application and *MxCuBE* acts as a server, communicating with DNA using XML-encoded messages through http. The data collection pipeline (DCP) mode of DNA allows multiple samples to be selected for evaluation and strategy calculation (Beteva *et al.*, 2006 ▶). In both single-shot and DCP modes the results of the DNA characterization are stored in ISPyB.

**Figure 6:**
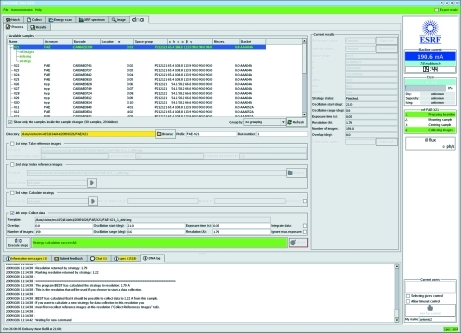
The DNA tab of the *MxCuBE* GUI. As in the Collect tab (Fig. 3 ▶) the sample area in the top part of the GUI shows information contained in the ISPyB LIMS (sample name, protein acronym, barcode, space group, unit-cell dimensions *etc.*) for the samples contained in the SC3 sample changer. Checkboxes are used to choose which tasks are to be carried out. Following successful characterization, unit-cell parameters and crystal point group are shown in the ‘current results’ brick. The data collection strategy calculated using *BEST* is displayed both in this brick and in the task area of the tab. Individual or a sequential series of tasks are carried out by clicking on ‘DNA execute steps’.

The use of multiple control interfaces causes confusion to infrequent users. Therefore, an interface to the DNA Expert System was developed and integrated into *MxCuBE*. This interface has its own tab (Fig. 6 ▶) and allows use of DNA in single-shot mode only. It is possible to execute a single action (*i.e.* taking reference images, indexing reference images, calculating a strategy, collecting/integrating/analysing a data set) or to chain several actions together. A sub-tab allows access to the html output provided by DNA including the results of auto-indexing, test image integration and data collection strategy calculation using *BEST* (Bourenkov & Popov, 2006 ▶). These results are also stored in, and accessible *via*, ISPyB.

### 3.5. *MxCuBE* features for remote access

As is the case at the SSRL MX facilities (González *et al.*, 2005 ▶) and as described elsewhere (Gabadinho *et al.*, 2008 ▶), the NoMachine *NX* software (http://www.nomachine.com/) was chosen to support remote access at the ESRF’s MX beamlines. This allows remote users to connect to a ESRF firewall computer from where they can connect and log in to the relevant beamline control computer and start *MxCuBE* and other beamline applications. These applications are displayed on the user’s local workstation from where the remote user can, when given permission, control the beamline.

A major concern when developing remote access was the requirement that users at the beamline and users elsewhere must be aware of each other and be able to communicate with each other. This implies several instances of *MxCuBE* running at the same time and that all instances are connected. Supporting multiple *MxCuBE* applications running concurrently was achieved by opening a server in the beamline instance of *MxCuBE* (the master instance) to which all other instances (slave instances) connect *via* a simple socket protocol (Fig. 1 ▶). Only one instance of the GUI can control the instrumentation at any one time. In the master instance of *MxCuBE* an account (with extra privileges) was created for beamline scientists and operators. This allows beamline staff to survey all operations carried out by remote slave instances of *MxCuBE* or, should the need arise, re-take control of the beamline without the explicit authorization of the remote user currently in control.

At the start of a remote-access experiment the beamline is controlled by ESRF staff logged in to *MxCuBE* *via* the operator account. During this phase of the experimental session, remote users can connect to the beamline control computer and start a slave instance of *MxCuBE* which, at first, has access only to a very limited subset of operations such as logging in. Once logged in, the only actions initially enabled are the sending and receiving of messages to other *MxCuBE* instances *via* the ‘Chat’ tab and the ability to request control of the beamline *via* the remote-control brick (Fig. 2 ▶). Remote users can only start their experiments when the beamline scientist, or operator, explicitly cedes control.

Slave instances of *MxCuBE* cannot directly take control from any other instance of the beamline GUI. However, they can view all other instances of *MxCuBE* running on a particular beamline and cede control to another slave instance of the GUI if this is requested. As the current state of *MxCuBE* on a particular beamline is mirrored across different instances logged into the same account, collaborative work has become popular. Geographically diverse researchers (*i.e.* different users in the same ESRF Block Allocation Group) can share beam time by passing control between *MxCuBE* instances. Although only one instance can physically control the beamline, others can monitor the experiment, give suggestions over the message feature, or request control of the beamline in order to perform experiments themselves. This mirroring of GUI instances is also useful for beamline staff during technical support, as they can log in to *MxCuBE* using the operator account to diagnose and resolve problems.

If during an experiment a remote user loses, for whatever reason, connection to *MxCuBE*, control reverts back to the beamline’s server-instance. In such cases, users must reconnect to *MxCuBE* and ask for control of the beamline. To avoid delays when beamline staff are not present (*i.e.* overnight) it is possible to set a timeout such that control passes to the requestor if not explicitly denied within 30 s. At the end of the experimental session, control users can return control to the beamline operator or scientist simply by logging out of *MxCuBE*.

## 4. Discussion

As has already been noted (McPhillips *et al.*, 2002 ▶; Arzt *et al.*, 2005 ▶), the development and implementation of standardized user-friendly software environments for the control of the increasingly complex hardware found at synchrotron beamlines for MX is a challenging task. This is particularly true at facilities such as the ESRF where the beamlines to be controlled have very different characteristics (*i.e.* fixed wavelength, variable wavelength, microfocus, MD2 or MD2M diffractometers). The *MxCuBE* GUI has profited from the flexibility that the ESRF BlissFramework applications provide. That these can be heavily and rapidly configurable through the use of the hardware repository allows the flexibility to construct a common interface whilst, at the same time, tuning control to a particular beamline’s instrumentation.

The first prototype versions of *MxCuBE* were deployed on the ESRF’s MX beamlines and the Collaborative Research Group (CRG) beamline BM14 in August 2005. Although still evolving as new technology becomes available and new ideas are implemented, the mature GUI is now also installed on the ESRF CRG beamline BM16 and has also been adopted (although with extensive changes in the interface) by MAX-lab beamline I911-3^1^ and BESSY beamline BL14.1^2^. Both these facilities are dedicated to experiments in MX. Preliminary discussions on the possible implementation of *MxCuBE* at data collection facilities at other synchrotron sources are also underway.

As should be evident, the *MxCuBE* interface is highly modular. New functionality can easily be incorporated and redundant behaviour easily removed. The modular nature of the interface also means that prototype tabs or bricks can be incorporated and tested on a single beamline’s version of the GUI before being deployed on the rest of the ESRF’s MX facilities. Major ongoing developments are: the incorporation of bricks in the Collect tab that will provide an interface to the EDNA (the successor to DNA) framework (Incardona *et al.*, 2009 ▶); the creation of tabs allowing the user-friendly set-up and performance of mesh and line scans; the control of the on-line dehydration device, HC1b (Sanchez-Weatherby *et al.*, 2009 ▶); and the addition of functionality allowing sample evaluation in the DCP (Data Collection Pipeline) mode currently only available *via* the DNA GUI. A heavily modified version of the system is available on the ESRF protein solution scattering beamline ID14-3^3^, where the flexibility of the BlissFramework has allowed the merging of generic features used for MX with the specific controls required for solution scattering.

While *MxCuBE* provides a visual framework for helping beamline users carry out MX experiments, the program also triggers other processes in a more transparent fashion. For example, *MxCuBE* automatically generates input files for the data processing programs *MOSFLM* and *XDS* if a data collection run contains four or more images contiguous in ϕ. In the near future, *MxCuBE* will automatically start a data integration and analysis pipeline which will launch, for each data set collected, one or more software packages in parallel. Information parsed by *MxCuBE* will then automatically be used to upload results of the data analysis into the ISPyB database. Further functionality will include the ability for *MxCuBE* to query and retrieve information directly from ISPyB. This information, including the results of sample ranking, the data collection strategies calculated using EDNA, crystal orientation or centring position, could subsequently be used for data collection.

## Footnotes

1
               http://cassiopeia.maxlab.lu.se/index/cms-filesystem-action?file=/Public/Cassiopeia%20Newsletter%20No%204.pdf.

2
               http://www.helmholtz-berlin.de/forschung/funkma/soft-matter/forschung/bessy-mx/beamlines/bl14-1/index_en.html.

3
               http://go.esrf.eu/bioSAXS.
